# Uncertainty Estimation in Unsupervised MR-CT Synthesis of Scoliotic Spines

**DOI:** 10.1109/OJEMB.2023.3262965

**Published:** 2023-03-29

**Authors:** Enamundram Naga Karthik, Farida Cheriet, Catherine Laporte

**Affiliations:** Department of Electrical EngineeringÉcole de technologie supérieure 14849 Montréal H3C 1K3 Canada; Institute of Biomedical EngineeringPolytechnique Montréal and Mila - Québec AI Institute 535330 Montréal H3C 3A7 Canada; Department of Computer Engineering and Software EngineeringPolytechnique Montréal 5596 Montréal H3T 1J4 Canada; CHU Sainte-Justine Research Center 25461 Montréal H3T 1C5 Canada; Department of Electrical EngineeringÉcole de technologie supérieure 14849 Montréal H3C 1K3 Canada; CHU Sainte-Justine Research Center 25461 Montréal H3T 1C5 Canada

**Keywords:** Bayesian Uncertainty, Generative Adversarial Networks, Scoliosis, Interpretability, Unsupervised Learning

## Abstract

Uncertainty estimations through approximate Bayesian inference provide interesting insights to deep neural networks' behavior. In unsupervised learning tasks, where expert labels are unavailable, it becomes ever more important to critique the model through uncertainties. This paper presents a proof-of-concept for generalizing the aleatoric and epistemic uncertainties in unsupervised MR-CT synthesis of scoliotic spines. A novel adaptation of the cycle-consistency constraint in CycleGAN is proposed such that the model predicts the aleatoric uncertainty maps in addition to the standard volume-to-volume translation between Magnetic Resonance (MR) and Computed Tomography (CT) data. Ablation experiments were performed to understand uncertainty estimation as an implicit regularizer and a measure of the model's confidence. The aleatoric uncertainty helps in distinguishing between the bone and soft-tissue regions in CT and MR data during translation, while the epistemic uncertainty provides interpretable information to the user for downstream tasks.

## I. Introduction

Scoliosis is a complex 3D deformity of the trunk involving lateral deviation in the spine and axial rotation of the vertebrae. Surgical treatment is necessary in severe cases. Magnetic resonance imaging (MRI) is a reliable and radiation free pre-operative imaging modality that can provide a 3D model of the spine, to which intra-operative images can be registered, provided that accurate segmentation of the vertebrae can be achieved. However, segmenting bones directly from MRI is difficult as it provides poor contrast for bone structures. On the other hand, bones are easily segmented in computed tomography (CT) images, but these are rarely acquired in the context of scoliosis due to the excessive radiation exposure. In preliminary work [1], we demonstrated the feasibility and accuracy of unsupervised scoliotic spine segmentation in MRI via intermediate pseudo-CT images generated through MR-CT synthesis using a deterministic CycleGAN model [2] trained on unpaired MR and CT spine data. This paper presents a novel Bayesian extension to this CycleGAN model that aims at increasing its interpretability by providing uncertainty estimates.

The interpretability of deep learning (DL) models is an important focus in recent literature [3], [4], [5], [6], [7]. However, these recent advances have not been translated to the healthcare domain. Inductive biases such as the presence of noise in the data and the implicit assumptions made by humans during data acquisition and manual annotation tend to go unnoticed. As a result, it is difficult to understand whether a model's performance is a true indication of the confidence in its predictions. Uncertainty quantification in DL models is one method of gaining nuanced insights into the models' behavior. The outputs of uncertainty-equipped models could be subsequently deployed in clinical settings for better diagnosis, follow-up and treatment [8].

Existing work focuses on uncertainty estimation in supervised learning problems (with labelled datasets), typically using Bayesian approximation and ensemble learning techniques [9]. There are two types of uncertainty that one can measure: (i) *epistemic*, which captures the uncertainty of the model over its parameters, and (ii) *aleatoric*, which captures the noise inherent in the data, such as the noise in the labels due to the inter-rater variability. Nair et al. [10] proposed voxel-based uncertainty measures using Monte Carlo Dropout [11] for 3D segmentation of multiple sclerosis lesions. Wang et al. [12] proposed a mathematical framework for estimating aleatoric uncertainty based on various data augmentation methods applied to brain MRI to understand the effect of these transformations of the input data on the segmentation outputs at test-time.

Recent advances in generative adversarial network (GAN)-based medical image synthesis [13], [14], [15], [16], [17] have shown great results in generating artificial images in different modalities that can be used as an nearly-identical proxies for subsequent downstream tasks (such as segmentation). Hemsley et al. [18] combined the estimation of aleatoric and epistemic uncertainties in supervised MR-CT synthesis of the brain using conditional GANs. Though a supervised method, theirs is the only work that addresses the importance of uncertainty quantification in medical image synthesis.

**Fig. 1.**
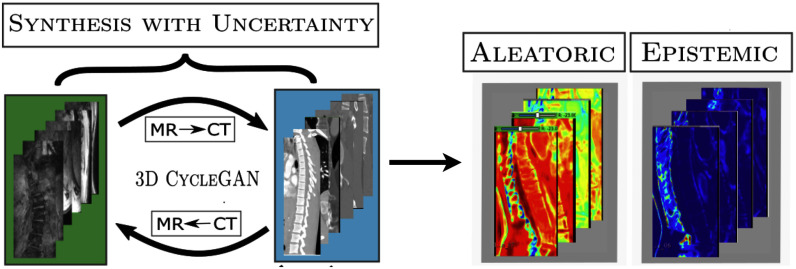
Workflow of our method. The proposed Bayesian adaptation of CycleGAN synthesizes both MR and CT volumes along with generating their aleatoric and epistemic uncertainty maps for improving model interpretability.

Our contributions in this paper are as follows:

1) We introduce a Bayesian adaptation of CycleGAN to estimate the aleatoric and epistemic uncertainties in addition to the volume-to-volume translation between MR and CT data of scoliotic spines. The novelty here lies in the generalization of these uncertainties to an unsupervised image synthesis task.
2) We demonstrate that estimating the aleatoric uncertainty by making the model predict the voxel-wise standard deviations in the loss function, acts as an implicit regularizer, thus helping the model improve its performance by learning to differentiate between the regions surrounding the spine. Furthermore, estimating the epistemic uncertainty provides additional interpretable information in terms of confidence maps (Fig. 1, right).
3) To improve translation across vertebral bone boundaries between the two modalities, we impose gradient correlation between the original and the synthesized volumes as an additional constraint (see II-C).

In summary, the proposed uncertainty estimation helps in offsetting the lack of external supervision by helping the model become self-sufficient and interpretable. The code is available at this link.^1^^1^https://github.com/naga-karthik/3D-CycleGAN-with-Uncertainty Fig. 1 shows a flowchart of our method. The rest of the paper is structured as follows: Section II describes our dataset and methodology. Section III presents ablation experiments demonstrating the contribution of uncertainty estimates and other model components towards the performance and interpretability of volume translation. Section IV discusses the implications of these results and Section V concludes our work.

## II. Methods and Materials

### A. Cycle-Consistent GANs

CycleGAN [2] is an image-to-image translation method that aims to tie two *unpaired* data domains $X$ and $Y$ together through adversarial training by synthesizing realistic images across these domains. Given two sets of unpaired training examples $\lbrace x_{i} \rbrace _{i=1}^{N} \in X$ and $\lbrace y_{j} \rbrace _{j=1}^{M} \in Y$, the model learns two function mappings simultaneously using two generators $G_{X \to Y}$ and $G_{Y \to X}$. Since voxel-wise comparison after synthesis is infeasible due to the unavailability of paired data, the cycle-consistency loss is introduced, which is defined as:

\begin{align*}
 \mathcal {L}_{\text {Cycle}} & = \mathbb{E}_{x \sim p_{\text {real}}(x)} [ || G_{\text {Y} \to \text {X}} (G_{\text {X} \to \text {Y}} (x)) - x ||_{1} ] \\
& \quad + \mathbb{E}_{y \sim p_{\text {real}}(y) } [ || G_{\text {X} \to \text {Y}} (G_{\text {Y} \to \text {X}} (y)) - y ||_{1} ], \tag{1}
\end{align*}

where $ || \cdot ||_{1}$ denotes the $L_{1}$-norm between the real and recovered samples for each domain and $p_{\text {real}}(x)$ and $p_{\text {real}}(y)$ denote the true data distributions from which inputs $x$ and $y$ are sampled [2].

### B. Bayesian Uncertainty Estimation in CycleGAN

This section describes our theoretical contribution: estimation of the aleatoric and epistemic uncertainties in the unsupervised CycleGAN model. Hereafter, the domains $X$ and $Y$ are denoted as MR and CT, respectively. Likewise, the generators $G_{X \to Y}$, $G_{Y \to X}$ and the discriminators $D_{X}, D_{Y}$ are denoted as $G_{MR \to CT}, G_{CT \to MR}$ and $D_{MR}, D_{CT}$, respectively. The real MR and synthesized CT volumes are denoted by $I_{MR}$ and ${\hat{I}_{SynCT}}$, and the real CT and synthesized MR volumes are denoted by $I_{CT}$ and ${\hat{I}_{SynMR}}$.

#### 1) Unsupervised Aleatoric Uncertainty

We propose a novel adaptation to the cycle-consistency loss that also extracts the heteroscedastic aleatoric uncertainty while being unsupervised. Recall from (1) that the cycle-consistency loss computes the $L_{1}$-norm between the recovered sample and the original input (real) sample. Therefore, the real sample acts as a pseudo-ground truth for the recovered sample so that its major characteristics, as approximated by the recovered sample, remain intact during consecutive forward and backward translations. Hence, we propose to compute the aleatoric uncertainty in CycleGAN as:

\begin{align*}
& \mathcal {L}_{\text {AleaCycle}} \\
 & = \! \left(\! \mathbb{E}_{x \sim I_{\text {MR}}} \left[ \frac{|| G_{\text {CT} \to \text {MR}} (G_{\text {MR} \to \text {CT}} (x)) - x ||_{1}}{\exp {(\log (\hat{\sigma _{x}})})} \right] + \frac{1}{2}\log (\hat{\sigma _{x}}) \!\right) \\
 & \quad + \! \left(\! \mathbb{E}_{y \sim I_{\text {CT}}} \left[ \frac{|| G_{\text {MR} \to \text {CT}} (G_{\text {CT} \to \text {MR}} (y)) - y ||_{1}}{\exp {(\log (\hat{\sigma _{y}})})} \right] + \frac{1}{2}\log (\hat{\sigma _{y}}) \! \right), \tag{2}
\end{align*}

where $\hat{\sigma _{x}}$ and $\hat{\sigma _{y}}$ are the predicted voxel-wise standard deviations of the MR and CT volumes respectively.

Thus, our proposal is to make the model predict the logarithm of the standard deviation of the real input sample, in addition to the recovered sample that is already being computed for the original cycle-consistency loss. We call this the *aleatoric cycle-consistency loss* $(\mathcal {L}_{\text {AleaCycle}})$. It must also be noted that predicting aleatoric uncertainty attenuates the loss function, in that the $\exp {(\log (\hat{\sigma })})$ term in the denominator tempers the residual $L_{1}$ loss in the numerator. For inputs resulting in high uncertainty, this term reduces its direct effect on the loss. Using $\log (\hat{\sigma })$ rather than $\hat{\sigma }$ ensures that the model does not predict high uncertainty for all inputs (thus ignoring the data), in which case it is penalized as the contribution from the $\log (\hat{\sigma })$ term increases.

#### 2) Epistemic Uncertainty

Epistemic uncertainty can be obtained by placing distributions over the weights of the neural network (NN) [19]. We used Monte-Carlo (MC) Dropout [11], a popular variational inference-based method. In practice, the network is trained with dropout applied before every weight layer. During inference, $T$ stochastic forward passes are performed through the network with dropout *enabled*, where $T$ is the number of MC samples. The mean and variance of these MC samples are then computed, resulting in the predictive mean and model uncertainty (predictive variance), respectively.

#### 3) Unifying Epistemic and Aleatoric Uncertainties

Let $\hat{I}_{SynMR}$ and $\hat{I}_{SynCT}$ be the synthesized MR and CT volumes, and $\log (\hat{\sigma }_{SynMR})$ and $ \log (\hat{\sigma }_{SynCT})$ be the predicted log standard deviations after translation. Instead of using (1), the updated aleatoric cycle-consistency (2) is used, where, in addition to the recovered MR and CT volumes, their log standard deviations are also learned implicitly. During inference, the model weights are sampled from the approximate posterior $\hat{w} \sim q_{\theta }^*(W)$ to obtain the synthesized volumes along with the aleatoric uncertainties as follows:

\begin{equation*}
\left[ \hat{I}_{SynCT}, \log (\hat{\sigma }_{SynCT}) \right] = G_{MR \to CT}^{\hat{w}}(I_{MR}) \tag{3}
\end{equation*}

where $G_{MR \to CT}$ is parameterized by the weights $\hat{w}$. Therefore, the output of a single generator provides both the synthetic volume and a measure of aleatoric uncertainty.

At test-time, each stochastic forward pass with weights $\lbrace \hat{w} \rbrace _{t=1}^{T}$ results in an unbiased estimate of the synthetic CT volume $ \lbrace \hat{I}_{SynCT}^{\; \hat{w}_{t}} \rbrace _{t=1}^{T}$ and the aleatoric uncertainty map $ \lbrace \log (\hat{\sigma }_{SynCT}^{\; \hat{w}_{t}}) \rbrace _{t=1}^{T}$. Then, the mean and variance of these $T$ stochastic forward passes are computed, which are the predictive mean (left) and model uncertainty (right), respectively. They are given by:

\begin{equation*}
\mathbb{E}\left[ \hat{I}_{SynCT}^{\;\hat{w}_{t}} \right] = \frac{1}{T} \sum _{t=1}^{T} \hat{I}_{SynCT}^{\; \hat{w}_{t}} \quad \text {and} \quad \text {Var}\left(\hat{I}_{SynCT}^{\; \hat{w}_{t}} \right). \tag{4}
\end{equation*}

Likewise, the final aleatoric uncertainty is:

\begin{equation*}
\mathbb{E} \left[ \log \left(\hat{\sigma }_{SynCT}^{\; \hat{w}_{t}} \right) \right] = \frac{1}{T} \sum _{t=1}^{T} \log \left(\hat{\sigma }_{SynCT}^{\; \hat{w}_{t}} \right). \tag{5}
\end{equation*}

### C. Gradient Consistency Loss

We also emphasize on the accurate translation of the bone boundaries during the artificial synthesis of the CT volumes. This is in order to facilitate a potential downstream task such as the segmentation of the vertebrae. Therefore, gradient correlation, defined as the normalized cross correlation between the gradients of two images, is introduced as an additional constraint [1], [14]. Given two volumes $A, B$, it is defined as:

\begin{align*}
 & \text {GradCorr}(A, B) = \frac{1}{3} (NCC (\nabla _{X} A, \nabla _{X} B) \\
& \quad + NCC (\nabla _{Y} A, \nabla _{Y} B) + NCC (\nabla _{Z} A, \nabla _{Z} B)), \tag{6}
\\
 \text{where }& NCC (\nabla A, \nabla B) \\
 & = \! \left(\frac{ \sum _{i,j} (\nabla A - \mu _{\nabla A}) (\nabla B - \mu _{\nabla B})}{ \sqrt{\sum _{i,j} (\nabla A - \mu _{\nabla A})^{2} } \sqrt{\sum _{i,j} (\nabla B - \mu _{\nabla B})^{2} } } \right), \tag{7}
\end{align*}

with $\nabla$ representing the gradients of the input volume in $X, Y$ and $Z$ directions. $\mu _{\nabla J}$ is the mean of the gradient of volume $J$. Therefore, gradient consistency (GC) loss is defined as:

\begin{align*}
 \mathcal {L}_{\text {GC}} & = \frac{1}{2} [ \mathbb{E}_{x \sim I_{\text {MR}}} (1 - \text {GradCorr}(x, G_{\text {MR} \to \text {CT}} (x))) \\
& \quad + \mathbb{E}_{y \sim I_{\text {CT}}} (1 - \text {GradCorr}(y, G_{\text {CT} \to \text {MR}} (y))) ]. \tag{8}
\end{align*}

From a Bayesian perspective, the GC constraint between the gradients of the MR and synthesized CT volumes can also be interpreted as a prior that is encoded into the model during training.

Lastly, as with all GANs, an adversarial loss is also defined to map the source data distribution to the target data distribution. For the mapping defined by $ G_{MR \to CT}: I_{\text {MR}} \to I_{\text {CT}}$ and its discriminator $ D_{CT}$, the objective is:

\begin{align*}
\mathcal {L}_{\text {CT}} = & \mathbb{E}_{y \sim I_{\text {CT}}} [ \log D_{\text {CT}}(y) ] \\
& + \mathbb{E}_{x \sim I_{\text {MR}}} [ \log (1 
 - D_{\text {CT}}(G_{\text {MR} \to \text {CT}}(x))) ]. \tag{9}
\end{align*}

Similarly, the objective for the reverse path is:

\begin{align*}
\mathcal {L}_{\text {MR}} = & \mathbb{E}_{x \sim I_{\text {MR}}} [ \log D_{\text {MR}}(x) ]\\
& + \mathbb{E}_{y \sim I_{\text {CT}}} [ \log (1 - D_{\text {MR}}(G_{\text {CT} \to \text {MR}}(y))) ], \tag{10}
\end{align*}

where $x$ and $y$ are the volumes from the MR and CT domains.

**Fig. 2.**
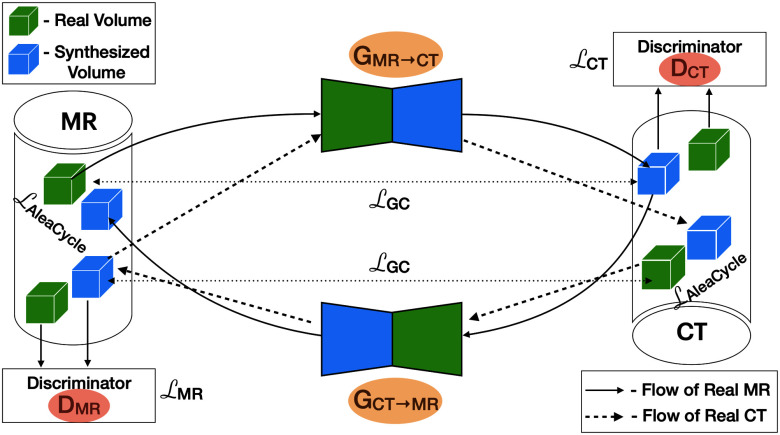
CycleGAN with 2 generators (orange) and 2 discriminators (red). Each cylinder represents an imaging modality with green boxes showing the real volumes and blue boxes showing the synthetic volumes of that domain. *Forward Cycle:* (solid arrows) Starting from green box (left, top), going through $G_{MR \rightarrow CT}$ to blue box (right, top), then going through $G_{CT \rightarrow MR}$ to recover blue box (left, top). *Backward Cycle:* (dashed arrows) Starting from green box (right, bottom), going through $G_{CT \rightarrow MR}$ to blue box (left, bottom), then going through $G_{MR \rightarrow CT}$ to recover blue box (right, bottom). The aleatoric cycle-consistency loss ($\mathcal {L}_{AleaCycle}$) is calculated between the real and recovered volumes (top left and bottom right). The gradient-consistency loss ($\mathcal {L_{GC}}$) is calculated between the real and synthesized volumes (top left, top right and bottom left, bottom right). Figure adapted from [14].

The full objective function to be optimized is thus:

\begin{align*}
& \left(G_{\text {CT} \to \text {MR}}^*, G_{\text {MR} \to \text {CT}}^* \right) \\
 & \quad = \arg \min _{ \underset{ G_{\text {CT} \to \text {MR}} }{ G_{\text {MR} \to \text {CT}} } } \max _{ \underset{ D_{\text {CT}} }{ D_{\text {MR}}} } (\mathcal {L}_{\text {CT}} + \mathcal {L}_{\text {MR}} + \lambda \mathcal {L}_{\text {AleaCycle}} + \gamma \mathcal {L}_{\text {GC}}), \tag{11}
\end{align*}

where $\lambda$ and $\gamma$ are the hyperparameters for weighting cycle- and GC losses. We set $\lambda =10.0$ and $\gamma = 0.5$ for the best results. Fig. 2 illustrates CycleGAN with our proposed uncertainty framework.

### D. Datasets

The MR and CT datasets were acquired from 3 different sources (2 for MR and 1 for CT). For MR, we used the dataset from the 2018 MICCAI Challenge on Automatic Intervertebral Disc Localization and Segmentation from 3D Multi-modality MR (M3) Images^2^^2^https://ivdm3seg.weebly.com/ consisting of 16 volumes of the lumbar spine, comprised of 4 mutually aligned MR modalities for studying the effect of prolonged bed rest on lumbar intervertebral discs. Our second source is a subset of the dataset described by Chevrefils et al. [20] consisting of MRI 3D multi-echo data volumes from 11 adolescent idiopathic scoliosis patients with deformities ranging from mild to severe, acquired from CHU Sainte-Justine in Montréal, Québec. This dataset focused on the thoracic region (T1-T12) of the spine. For CT, we used 2 sample volumes provided by 3D Slicer. The supplementary material describes the preprocessing, data augmentation methods and for a few sample images from the dataset.

### E. Training Details

**Fig. 3.**
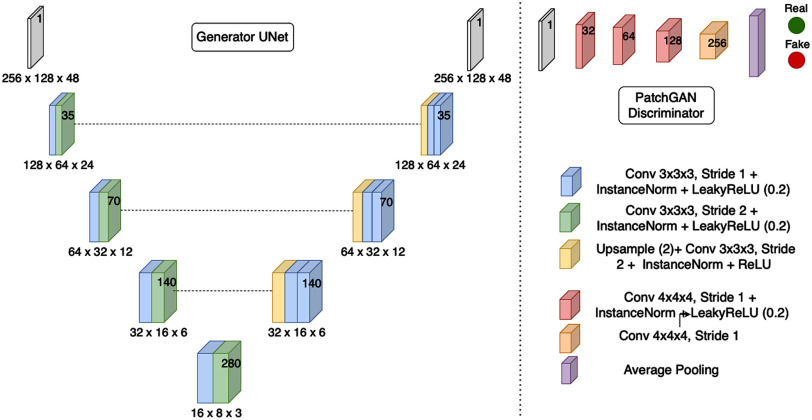
Generator and discriminator architectures used. Numbers inside each block represent the feature maps at that resolution.

A 3D UNet [21] was used as the generator network and PatchGAN [2] was used as the discriminator network. Fig. 3 illustrates the model architecture. Adam optimizer [22] was used with batch size 2 and learning rate of 0.0002. The model was trained for 200 epochs with linearly decaying the learning rate after the first 100 epochs. Training time was 93 hours on 4 NVIDIA Tesla V100 GPUs with 32 GB memory. However, the inference time was less than 5 seconds on a single NVIDIA 1080Ti 12 GB GPU. Full description of the model architecture can be found in the supplementary material.

## III. Experiments and Results

Five scoliotic patients were used in the test set, hereafter referred to as *Patient1 (P1)*, *Patient3 (P3)*, *Patient4 (P4)*, *Patient11 (P11)*, and *Patient12 (P12)*. Their spinal deformities range from mild (Cobb $12^\circ -24^\circ$) to severe (Cobb $43^\circ -60^\circ$). The accuracy of vertebral bone segmentation using the CT image translations resulting from the proposed method, including uncertainty estimation, was found to be similar to the CycleGAN model without uncertainty discussed in our preliminary work [1]. These quantitative results are available in the supplementary material. The remainder of this section instead focuses on the experiments conducted to gain a qualitative understanding of the effects and interactions between the novel uncertainty estimations and gradient consistency (GC) loss towards the quality and interpretability of the translated CT volumes. The quality of the translations can be evaluated by observing the similarity between the shapes of the bone structures depicted in both MR and the synthesized CT volumes. As soft tissues are not clearly represented in CT as they are in MR, one would expect high aleatoric uncertainty in such regions of the synthesized CT volumes. Likewise, epistemic uncertainty is also expected to be relatively higher at the bone boundaries, especially due to the difficulty in translating the partial volume effects in MRIs.

Since the GC loss and uncertainty estimations are the two main additions to the CycleGAN architecture, we conducted an ablation study where all four combinations concerning those two modifications were considered. The purpose of these experiments are two-fold: (1) to understand the benefits of using uncertainty estimates thereby leading to informed interpretations of the model's predictions, and (2) to visualize the effects of the gradient consistency constraint specific to MR-CT synthesis. Hereafter, we refer to “soft” prediction as the mean of $T$ MC samples (here, $T=20$) and “hard” prediction as the output resulting from only one set of (best) weights.

### A. Effect of the GC Loss Without Uncertainty

**Fig. 4.**
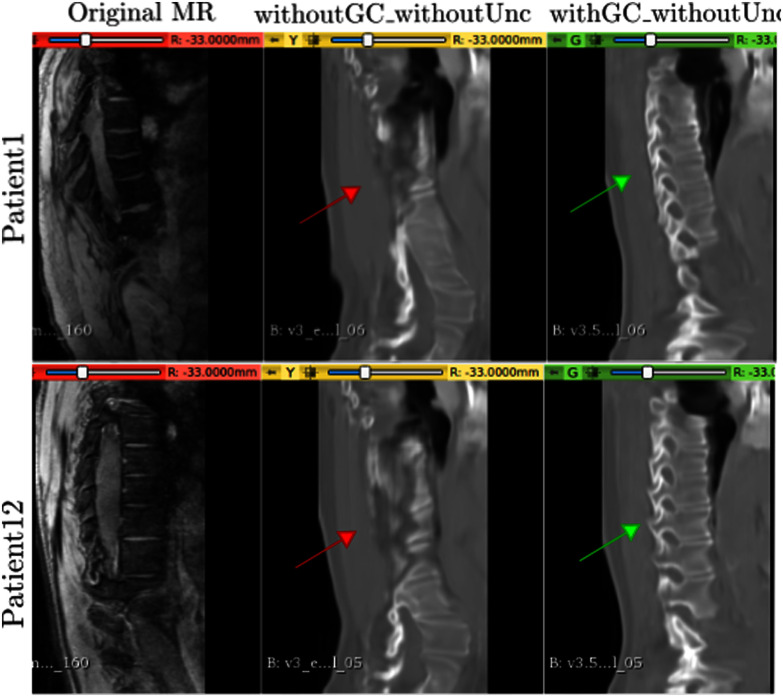
Hard Synthesized CT predictions for P1 and P12. Left-to-right: Original MR slice, synthesized CTs without uncertainty for models without and with GC, respectively. Red and green arrows show the difference in translation without and with GC. Unc.$=$ Uncertainty.

This subsection compares the results of: (i) the model trained without GC loss and without uncertainty computations (i.e. the default CycleGAN) (“withoutGC_withoutUnc”), and (ii) the model trained with GC loss but without the uncertainties (“withGC_withoutUnc”), described in our preliminary work [1]. Fig. 4 shows the (hard) translations obtained for Patient1 and Patient12.

Considering the red and green arrows in Fig. 4, it is clear that optimizing for gradient consistency during training helps the model learn the vertebral shapes and localize the bone structures from the training volumes. However, the lack of uncertainty gives no estimate of the model's confidence, which can be useful for the downstream post-processing tasks such as segmentation.

### B. Effect of the GC Loss With Uncertainty

**Fig. 5.**
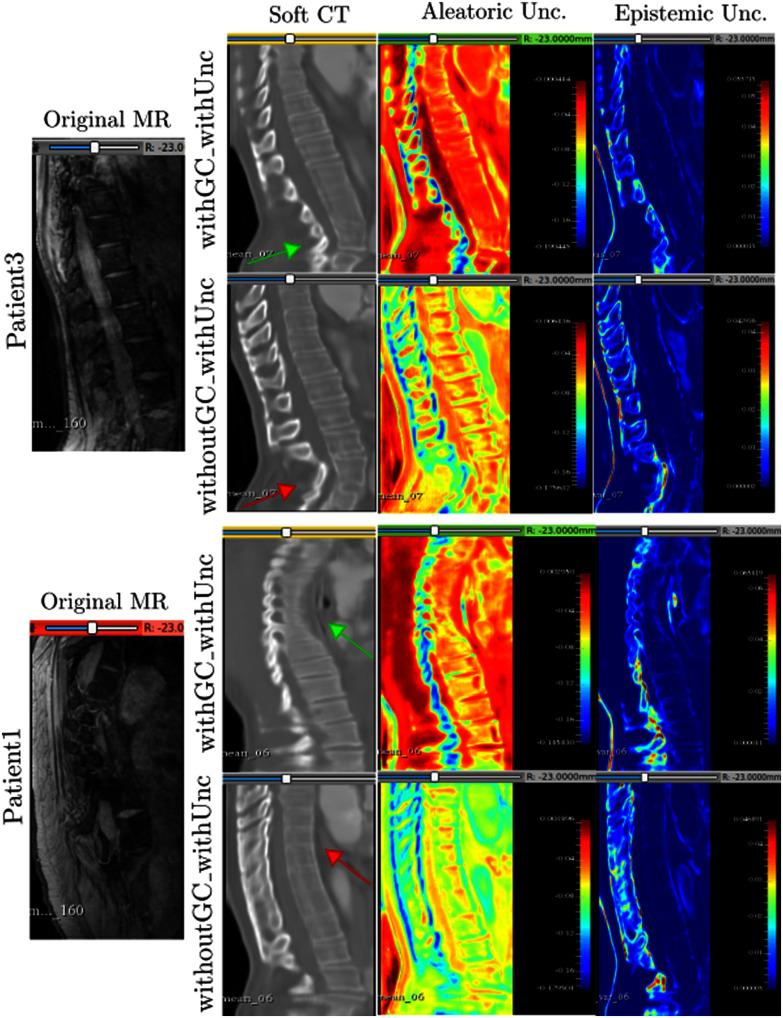
The soft translations along with aleatoric and epistemic uncertainties. Left-to-right: original sagittal MR slices for Patient1 and Patient3, “soft” CT predictions, aleatoric maps learned by the models, and epistemic uncertainties. Top-to-bottom: 1st and 3rd rows show results with GC, 2nd and 4th rows show results without GC. Green arrow points to the spinal curvature better translated with GC and red arrow shows the same region translated without GC. Blue and red regions in the uncertainty maps refer to low and high uncertainties respectively. “Unc.”$=$ Uncertainty.

This subsection compares the results of: (i) the model trained with both the GC loss and uncertainty enabled (“withGC_withUnc”), and (ii) the model trained without the GC loss but with uncertainty estimations (“withoutGC_withUnc”). Fig. 5 shows the translations and the uncertainty estimates for Patient1 and Patient3.

The bottom-half of Fig. 5 shows that the spinal curvature of Patient1 has been slightly better captured by the model that was trained with the GC loss (shown by green and red arrows). For Patient3 (top-half of Fig. 5), the bottom thoracic vertebrae have been better translated with GC (row 1) compared to the model trained without the GC loss (row 2).

Regarding the uncertainty maps, recall from (2) that the aleatoric uncertainty maps are learned by comparing the recovered MR volumes with the original ones. To satisfy cycle-consistency, the recovered MR volumes are solely based on the quality of the synthetic CT volumes from the forward cycle where the soft-tissue information is lost and bone structures are emphasized. Therefore, the high uncertainty corresponds to the soft tissue regions lost during the forward cycle translation going from MR to CT. Notice that the soft tissue regions in rows 1 and 3 (withGC) are fully red (highly uncertain), whereas the bones are in yellow and blue (relatively less uncertain). On the other hand, since the epistemic uncertainty depends only on the model parameters, it specifically shows that the model's confidence is low in translating the spinous processes.

In the case without GC (rows 2 and 4), the model was unable to distinguish between the bones and the soft tissues, hence predicting similar aleatoric uncertainty (yellow/green regions) across the entire image.

It must also be noted that by the virtue of estimating the aleatoric uncertainty, the model learns to distinguish between the bone and soft tissue regions by itself without any external conditioning.

### C. Effect of Modelling Uncertainty With GC Loss

**Fig. 6.**
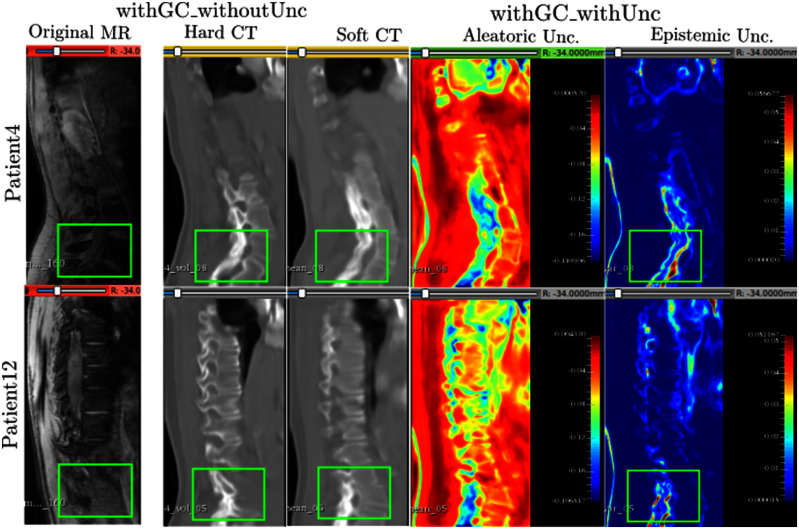
The hard and soft CT translations along with aleatoric and epistemic uncertainties for the latter. Left-to-right: Original MR slices, hard CT results for the model without uncertainty, columns 2, 3, and 4 - mean prediction with aleatoric and epistemic maps, respectively. Green boxes show the specific regions compared across translations. Blue and red regions in the uncertainty maps refer to low and high uncertainties respectively. “Unc” - Uncertainty.

This subsection compares the results of: (i) the model trained with the GC loss but without the uncertainties (withGC_withoutUnc), and (ii) the model trained with both the GC loss and uncertainty estimations (withGC_withUnc). Fig. 6 shows the hard and soft translations along with uncertainty estimations for Patient4 and Patient12.

Considering the green boxes across all slices in Patient4 and Patient12, the translation from the MR slice has accurately translated the spinous processes. The hard and soft predictions are similar to each other. However, the model trained with both GC and uncertainty conveys that it is not confident about its translation of the spinous processes. This appears in the form of high epistemic uncertainty within the green boxes. Therefore, this region requires supervision from the user during post-processing or the downstream segmentation task.

### D. Effect of Modelling Uncertainty Without GC Loss

**Fig. 7.**
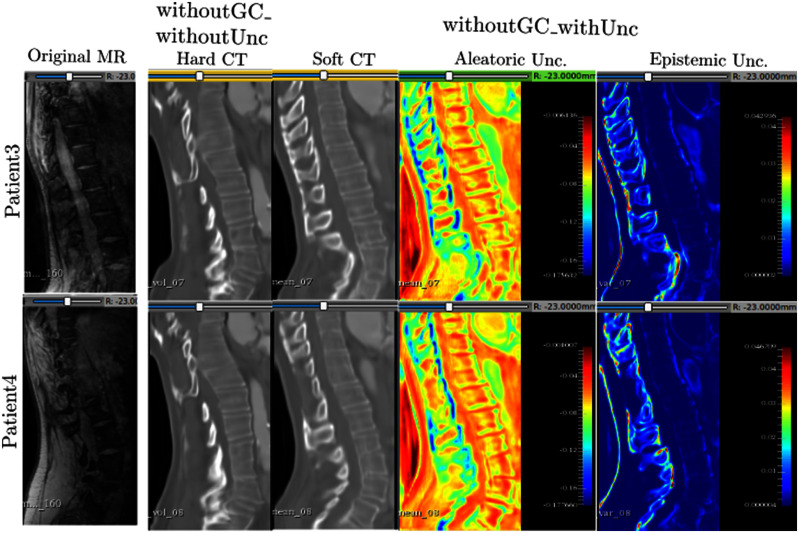
The hard and soft CT translations along with aleatoric and epistemic uncertainties for the latter. Left-to-right: Original MR slices, results for the model without uncertainty, columns 2, 3, and 4 - mean prediction with aleatoric and epistemic maps, respectively. Blue and red regions in the uncertainty maps refer to low and high uncertainties respectively. Unc. - Uncertainty.

This subsection compares the results of: (i) the model trained without the GC loss and without uncertainty estimations (“withoutGC_withoutUnc”) and, (ii) the model trained without the GC loss but providing uncertainty estimations (“withoutGC_withUnc”). Fig. 7 shows the corresponding results for Patient3 and Patient4.

The hard CT translations appear similar between patients for the model trained without GC and without uncertainty. However, due to the absence of uncertainty information, it is difficult to understand where the model might have translated incorrectly. While the soft translations themselves are not perfect, they are able to better capture the shapes of the spinous processes. In addition, depriving the model of GC and uncertainty constraints has affected its ability to learn the vertebral structure specific to the patient and output generic translations unlike its soft CT counterpart. Therefore, by modelling aleatoric uncertainty during training, the model tends to offset the lack of GC.

## IV. Discussion

Our experiments show how the GC loss and uncertainty estimations play a key role during and after training in the quality of the synthesized CT volumes. In Fig. 5 (experiments “withGC_withUnc” and “withoutGC_withUnc”), by the virtue of optimizing for GC, the model could automatically distinguish between the bones and the soft tissue regions (as shown with yellow and red regions of aleatoric uncertainty respectively). The corresponding epistemic uncertainty results specifically show high uncertainty in the spinous processes, thereby making them a target requiring increased supervision for downstream tasks. This leads to two more observations: (i) despite minor differences in the translations, it is better to optimize for the GC loss, in addition to modeling the uncertainty estimates, as long the training remains stable and memory constraints allow, and (ii) out of the two uncertainty maps, providing the user only with epistemic uncertainty is more useful for post-processing tasks, while the aleatoric uncertainty helps the model identify and distinguish different regions in the training data, leading to improved performance. These observations reinforce the idea that uncertainty estimations help extract more information from the unsupervised CycleGAN model. Lastly, the proposed uncertainty estimates, in turn, also benefit from the prior imposed by the GC constraint. It assumes that the underlying physical properties of the spine are sufficiently similar across the MR and CT data, which is a reasonable assumption as they belong to the same patient.

There are a few limitations to our work. First, out of the two uncertainties, only the epistemic uncertainty can be meaningfully interpreted by the end user as these are generated during test-time (we show in the supplementary material that the regions of high epistemic uncertainty helped in guiding the semi-automatic segmentation of the vertebral bodies). This is because the aleatoric uncertainty is typically obtained by comparing the model prediction with the actual ground truth, which is unavailable. To circumvent the lack of ground truth CT data, our method compared the MR volume recovered from the synthetized CT volume with the original MR volume. The aleatoric uncertainty map tends to capture the loss of soft tissue information that occurs during the forward MR-to-CT translation by acting as an implicit regularizer during training, which is not easily interpreted by the user. Second, due to the unavailability of expert-annotated vertebral labels in scoliotic CT data, it is difficult to quantitatively measure the benefit of uncertainty estimations.

## V. Conclusion

Our experimental results suggest that modelling uncertainties helps improve the unpaired translations while also providing interpretable confidence maps towards understanding the model's predictions. This constitutes a novel proof-of-concept towards the generalization of uncertainty estimation to unsupervised image synthesis problems.
